# Assessment of the Radiological Health Risk Due to the ^226^Ra Content in Drinking Water from the Calabria Region, Southern Italy

**DOI:** 10.3390/ijerph19169829

**Published:** 2022-08-09

**Authors:** Francesco Caridi, Giuseppe Paladini, Sebastiano Ettore Spoto, Santina Marguccio, Maurizio D’Agostino, Alberto Belvedere, Vincenza Crupi, Valentina Venuti, Domenico Majolino

**Affiliations:** 1 Dipartimento di Scienze Matematiche e Informatiche, Scienze Fisiche e Scienze della Terra, Università degli Studi di Messina, Viale Ferdinando Stagno D’Alcontres, 31-98166 Messina, Italy; 2 Dipartimento di Reggio Calabria, Agenzia Regionale per la Protezione dell’Ambiente della Calabria (ARPACal), Via Troncovito SNC, 89135 Reggio Calabria, Italy

**Keywords:** radium, drinking water, ingestion, radiological risk, dose

## Abstract

In this article, the authors report experimental results obtained for the assessment of the ^226^Ra content in 80 drinking water samples from the Calabria region, Southern Italy. The activity concentration, measured with the Perkin Elmer Tricarb 4910 TR Liquid Scintillation Counter (LSC) setup, was compared with the reference values reported in the Italian Legislative Decree 28/2016 in order to evaluate any possible radiological health hazards for the population in terms of ^226^Ra content due to the ingestion of the investigated drinking water. The obtained results put in evidence that the average ^226^Ra specific activity is lower than the LSC minimum detectable activity (MDA) in all cases, thus, excluding any radiological risk. They also represent the main reference for the investigated area and can be used as a baseline to extend this investigation to the whole region.

## 1. Introduction

Water used for drinking purposes normally contains radioactivity of natural origin, i.e., uranium (^238^U and ^235^U) and thorium (^232^Th) decay chain products and ^40^K, whose presence is mainly due to erosion phenomena of the rocks with which it comes into contact [1,2]. Water, especially when of underground origin, is, thus, enriched with the constituent elements of rocks, including radioactive ones [3,4,5]. Groundwater is normally not affected by natural and artificial radionuclides of anthropogenic origin, such as ^137^Cs [6]; however, these contributions can affect the superficial water that can often be used for drinking purposes [7]. Water controls are, therefore, aimed at looking for the presence of both artificial and natural radioactive substances [8,9].

The presence of natural radioisotopes in Italian drinking water as a hazard factor to the public is addressed today in detail by the national regulation currently in force, i.e., the Legislative Decree 28/2016, that implements the Directive 2013/51/Euratom. The Legislative Decree 28/2016 establishes the principles, criteria, and methods for radioactivity controls in water for human use [10]. In detail, it introduces indicator parameters and the related parameter values, i.e., Indicative Dose (D_ind_, 0.1 mSv y^−1^), ^222^Rn specific activity (100 Bq L^−1^), and ^3^H activity concentration (100 Bq L^−1^). It also establishes that the determination of the D_ind_ must always be carried out after a preliminary screening consisting of a gross alpha and beta specific activity assessment. In particular, in the case of gross alpha and gross beta specific activities higher than 0.1 Bq L^−1^ and 0.5 Bq L^−1^, respectively, the D_ind_ value could exceed the parameter value of 0.1 mSv y^−1^ set by the Italian legislation [10]. As a consequence, an analytical study must be carried out in order to calculate the D_ind_ based on the evaluation of both the specific activity of the radionuclides in the investigated samples (including ^226^Ra) and the dose coefficients reported in [11].

In the present study, a campaign of measurements was conducted with the aim of monitoring the ^226^Ra activity concentration in drinking water coming from public supplies in the Calabria region, Southern Italy. In detail, six sampling sites in the Reggio Calabria district, where only the gross alpha activity concentration exceeded the parameter value [10], were selected. Such investigation was aimed, on one side, at the development of a database concerning the presence of ^226^Ra in drinking water in the Calabria region, and, on the other side, at the evaluation of the associated radiological health risks for the population [12]. This communication supplements previous works referring to the monitoring of only the gross alpha and beta specific activities of Calabrian waters [2], providing original data on ^226^Ra, which is the most radiotoxic nuclide that contributes to the gross alpha activity concentration. Moreover, it complements previous studies referring to the ^222^Rn content in Calabrian drinking water [6,13], putting in evidence that the detected ^222^Rn concentration was mainly due to the radon gas emanating from rocks forming the aquifer rather than to the ^226^Ra decay.

Worth of note, the aforementioned approach appears to be crucial since ^226^Ra exposure can cause, on one side, detrimental effects on the human health, such as broken teeth, anemia, and cataracts, for high levels of exposure. On the other side, somatic effects can also occur, i.e., bone, cranial, and nasal tumors, being ^226^Ra characterized by high radiotoxicity and a chemical affinity with calcium [14,15].

## 2. Materials and Methods

**Figure 1:**
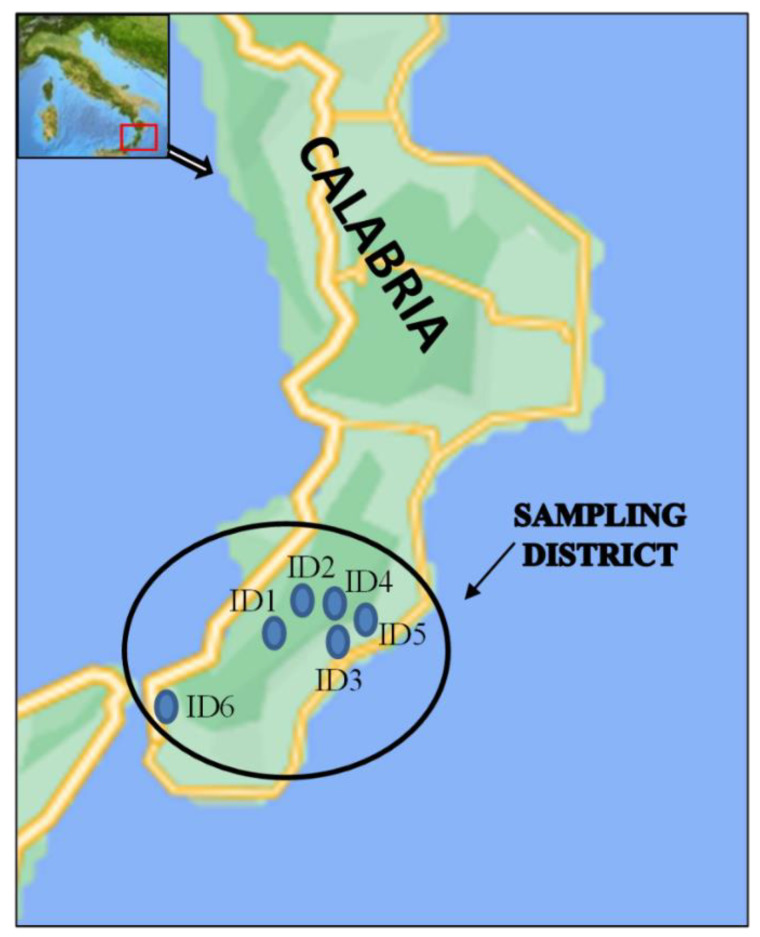
A map of the Calabria region, with the sampling district and the site IDs indicated.

Six different municipalities (ID#, # = 1, …, 6; see Table 1), located in the Calabrian district of Reggio Calabria (Figure 1), with springs, wells, and related fountains and tanks, were identified and monitored four times during the year 2021, one for each different season, according to what was reported in [10].

The sample collection was conducted based on the local weather conditions, which in some periods put severe limitations on the access to the sampling stations. A total of 80 drinking water samples were collected into 1 L of acidified polyethylene holders.

A Perkin Elmer Tricarb 4910 TR Liquid Scintillation Counter (LSC), with a 0–2 MeV (β particles) and a 0–10 MeV (α particles) energy range, a minimum acceptable efficiency of 60% for ^3^H (0–18.6 keV) and 95% for ^14^C (0–156 keV), an average background of 17 CPM for ^3^H and 26 CPM for ^14^C, and an external standard ^133^Ba, was employed for the measurements [2].

An initial volume of 200 mL of sample, acidified with 20 µL of 14 M ultrapure HNO_3_, was preconcentrated at a final volume of 20 mL and a pH of ~1.85. Of this total volume, only 10 mL were taken and mixed with 10 mL of Perkin Elmer Opti-Fluor scintillation cocktail immiscible in water, stored in a 20 mL plastic vial in a dark place, and, after a rest time of at least 23 days, counted for 1000 min, together with a blank sample of distilled water, used as background, for the ^226^Ra detection [16].

The ^226^Ra activity concentration (Bq L^−1^) was calculated using the following formula:

(1) $$C=\frac{N}{\varepsilon tV}$$

where *N* indicates the net counts, *ε* the counting efficiency, *V* the sample volume (L), and *t* the live time (s).

The quality of the experimental results was certified by the Italian Accreditation Body (ACCREDIA) [17].

In the selected sampling sites, where the gross alpha activity concentration was exceeded [18], the Indicative Dose was evaluated considering that when $\overset{n}{\underset{i=1}{\sum }}C_{i}\left(mis.\right)/C_{i}\left(der.\right)\leq 0.1,\;$ with *C_i_(mis.)* and *C_i_(der.)*, accounting for the measured and derived specific activities of the i-th radionuclide that contributed to the Indicative Dose, respectively, the D_ind_ value is lower than the parameter one [10]. For ^226^Ra, the derived specific activity is 0.5 Bq L^−1^, as reported in [10].

## 3. Results and Discussion

Table 2 reports the average ^226^Ra activity concentrations in the analyzed samples for each sampling site.

It can be noticed that all obtained average values are lower than the LSC minimum detectable activity (0.04 Bq L^−1^) [16] and are much lower than the ^226^Ra derived activity concentration (0.5 Bq L^−1^). It should be underlined that the analytical sensitivity of the measurements is completely adequate to the requirements of the Legislative Decree 28/2016.

Noteworthy, since up to now, there has not been enough information regarding the concentrations of ^226^Ra in drinking water, these results represent a main reference for the investigated area and can be used, in principle, as a baseline to extend this investigation to the whole region.

From a radiological point of view, the obtained results exclude a significant radioactive contamination of ^226^Ra for all the investigated samples and any possible radiological hazards for the population in terms of the ^226^Ra radioactive content [13].

It is important to put in evidence that a complete evaluation of the radiological health risks implies the calculation of the Indicative Dose after the specific activity quantification of all the relevant α-emitting radionuclides [10]. This would, therefore, involve a quantitative measurement of the activity concentration of the radioisotopes of Uranium, in particular ^234^U and ^238^U, and of Polonium, in particular ^210^Po, other than ^226^Ra, as widely reported in the literature [19,20,21].

Going on, the content of ^226^Ra in aquifers is not only related to the abundance of its parent radionuclides [22], but also it is controlled by sorption, desorption, and ion exchange processes [23]. In particular, factors related to geology and climate that affect the acidity, redox potential, degree of mineralization, and composition of groundwater, as well as their potential residence time, can also affect sorption and, thereby, the distribution pattern of Ra-isotopes [23]. Moreover, it is worth noting is that the investigated area falls within the Aspromonte Massifs, a geological segment of the Calabrian Arc, which consists of a series of Palaeozoic plutonic–metamorphic nappes, locally overlain by a Mesozoic–Cenozoic sedimentary cover. Accordingly, based on published cartography and several research studies [24,25], heterogeneous crystalline–metamorphic rocks, rich in U and Th, crop out in the area surrounding the sampling sites [26]. These rocks are affected by weathering processes, which are responsible for their chemical and mechanical transformation when interacting with the atmosphere, the hydrosphere, and the biosphere [27]. Furthermore, weathering plays a crucial role in controlling the chemical and mineralogical features of the final sedimentary products (clays, mineral oxides, oxyhydroxides, etc.), and, according to [28], the alteration products of the parent rocks located in the surrounding areas of the investigated sites are controlled by the general climate of the region (temperate and moderately humid). Finally, even if the obtained results were achieved following the official institutional indications [10], it has to be underlined that a periodical screening could be necessary in order to guarantee the safety of the drinking water for the sake of public health [29].

## 4. Conclusions

This article reports on the results of ^226^Ra specific activity measurements performed on drinking water samples coming from six different sampling sites in the Calabria region, Southern Italy, with the aim of evaluating the radiological health risk associated with ^226^Ra ingestion in terms of the ^226^Ra content. The investigation was conducted during the year 2021 by collecting 80 water samples from springs, wells, and related fountains and tanks.

The ^226^Ra activity concentration was measured by means of the Perkin Elmer Tricarb 4910 TR Liquid Scintillation Counter (LSC). The obtained results show that the ^226^Ra specific activities for all of the investigated drinking water are lower than the LSC minimum detectable activity. Therefore, it can be argued that the analyzed samples are safe for drinking and other domestic purposes in terms of ^226^Ra content, and, hence, no remedial actions are needed. The obtained results also represent a main reference for the investigated area and can be used as a baseline to extend this investigation to the whole region.

## Figures and Tables

**Table 1:** The investigated municipalities.

Site ID	Municipality
1	Cittanova
2	Giffone
3	Gioiosa Jonica
4	Mammola
5	Martone
6	Reggio Calabria

**Table 2:** The average ^226^Ra activity concentrations in the analyzed samples for each sampling site.

Site ID	C_Ra-226_ (Bq L^−1^)
1	<0.04
2	<0.04
3	<0.04
4	<0.04
5	<0.04
6	<0.04

## Data Availability

Not applicable.
